# *ADH1B*, *ADH1B/C* and *CYP2E1* Gene Polymorphism and the Risk of Fetal Alcohol Spectrum Disorder

**DOI:** 10.3390/genes14071392

**Published:** 2023-07-02

**Authors:** Arnold Kukowka, Bogusław Brzuchalski, Mateusz Kurzawski, Damian Malinowski, Monika Anna Białecka

**Affiliations:** 1 Department of Pharmacokinetics and Therapeutic Drug Monitoring, Pomeranian Medical University, Aleja Powstanców Wielkopolskich 72 St., 70-111 Szczecin, Polanddamian.malinowski@pum.edu.pl (D.M.);; 2 Department of Experimental and Clinical Pharmacology, Pomeranian Medical University, Aleja Powstanców Wielkopolskich 72 St., 70-111 Szczecin, Poland; mateusz.kurzawski@pum.edu.pl

**Keywords:** FASD, alcohol, SNP

## Abstract

Increasing alcohol consumption by women of childbearing age contributes to more frequent cases of fetal alcohol spectrum disorder. The cause of the syndrome is fetal alcohol exposure, particularly what is referred to as high prenatal alcohol exposure. Low metabolic activity of fetal enzymes shifts the burden of ethanol removal to maternal metabolism. One of the factors influencing the pathogenesis of FASD is the genetic background. It can determine the rate of elimination of ethanol, thus increasing or decreasing the time of fetal exposure to ethanol and also decreasing its concentration. Genetic polymorphisms could potentially play a significant role in these processes. In the present study, we considered three polymorphisms of genes implicated in the synthesis of enzymes involved in ethanol metabolism, i.e., *ADH1b* (rs1229984), *ADH1b/c* (rs1789891), and *CYP2E1* (rs3813867). The studied group consisted of 303 children and 251 mothers. Both mothers’ and children’s genotypes were considered in our analysis. There were no statistically significant differences between the respective groups of genotypes of the studied polymorphisms. However, the genetic background of FASD is still elusive.

## 1. Introduction

With the knowledge of the human genome, the opportunity has come to better understand the influence of genotype on phenotype in the context of central nervous system disorders. Single nucleotide polymorphisms (SNPs) were of particular interest to the researchers [1]. In diseases of multifactorial pathogenesis, such as epilepsy, schizophrenia, depression, or autism, knowledge of SNPs provides an opportunity to better understand the cause of the disease and to design more effective pharmacotherapy [2]. One of the disorders whose phenotypic presentation may be influenced by a genetic factor is fetal alcohol spectrum disorder (FASD) [3,4]. By definition, FASD includes fetal alcohol syndrome (FAS) and partial fetal alcohol syndrome (p-FAS), as well as alcohol-related birth defects (ARBD), alcohol-related neurodevelopmental disorders (ARND), and neurobehavioral disorders associated with prenatal alcohol exposure (ND-PAE) [5]. Epidemiologically, it is estimated that about 10% of pregnancies have prenatal alcohol exposure (PAE)—resulting in 77 children diagnosed with FASD and about 15 with the most severe form of FAS (fetal alcohol syndrome) for every 1000 newborns [6,7]. All developing fetal systems are susceptible to the harmful effects of ethyl alcohol [8], but the nervous system is the most vulnerable. It is primarily damaged by increased oxidative stress and harmful ethanol metabolites [9,10]. The alcohol toxicity results in a very extensive symptomatology of FASD, including symptoms such as dysmorphologies (e.g., short palpebral fissure, smooth philtrum, or thin upper lip), neuropsychological and sensory disorders, as well as neurological symptoms, including epilepsy [11,12]. 

Fetal exposure to alcohol is the only necessary condition for the development of FASD. However, not every fetus exposed to alcohol will later develop FASD—studies estimate that 10% to 15% of children exposed as fetuses to alcohol will suffer from FASD [13]. This is due to numerous other risk factors. Among these, we can distinguish three main groups of risk factors. Those dependent on the mother (e.g., mother’s age), those dependent on agent exposure (e.g., binge drinking or drinking outside of meals), and those dependent on the environment (e.g., little knowledge of FASD) [14]. Their role and the genetic influence are associated with the degree of risk for disorders on the spectrum of fetal alcohol disorders and their severity [3,15,16]. The variety of risk factors and the individual susceptibility to develop FASD prompt further research including genetic research. Individual susceptibility to alcohol toxicity, and thus the development of FASD, may be related to genetic determinants [3,17,18]. Particularly important is the activity of enzymes responsible for alcohol metabolism in both mothers and fetuses with h-PAE (high prenatal alcohol exposure) [17]. Two pathways of alcohol metabolism are distinguished: oxidative—involving alcohol dehydrogenase (ADH), aldehyde dehydrogenase (ALDH), catalase, and the P450 cytochrome (mainly CYP2E1)—and non-oxidative [18,19,20]. Both lead to the synthesis of products that are toxic [21]. For example, increased susceptibility to alcohol toxicity in the prenatal period may be associated with maternal genetic variants that determine the activity of enzymes involved in alcohol metabolism since they play a dominant role in the elimination of alcohol [3,13,17,18,22]. We distinguish three main genes that encode the activity of alcohol dehydrogenase (ADH)—*ADH1A*, *ADH1B*, and *ADH1C* [23]. The translation of these genes produces corresponding polypeptides, which are the subunits of the alcohol dehydrogenase isoenzymes. The translation product of the *ADH1B* gene is the β subunit [24]. One of the most studied polymorphisms of the *ADH1B* gene is ADH1B*2 (rs1229984). In the carriers, arginine at position 48 is converted to histidine (Arg48His) resulting in altered enzyme activity and alcohol metabolism [19]. Yin et al. found in an in vitro study that the maximum rate of oxidation of ethanol to acetaldehyde is about 100 times higher in carriers of the A allele. These individuals have β2 subunits that are components of aldehyde dehydrogenase isoenzymes [25]. Carriers of one or two A alleles have increased enzymatic activity of alcohol dehydrogenase—thus, the negative effects of alcohol consumption are present more quickly, which is associated with a lower risk of alcohol dependence [26,27,28]. Another less investigated SNP is rs1789891. It is located on the long arm of chromosome 4 between the *ADH1B* and *ADH1C* genes [29]. The role of this polymorphism is yet to be fully understood, but it is in total linkage disequilibrium with another polymorphism, rs1693482 in the *ADH1C* gene [29,30]. Rs1693482 results in a functional change, i.e., substitution of glutamine for arginine at position 272. People with the arginine variant at position 272 (C allele, 272Arg) metabolize ethyl alcohol twice as fast, and the enzyme itself has twice the affinity for ethanol compared to 272Gln (T allele, 272Gln) [31,32]. As shown in the study by Osier et al., the C allele is associated with a lower risk of alcoholism in the Asian population [33], which has also been confirmed in other studies with regard to this population [34,35]. A slower metabolism of ethyl alcohol was associated with the T allele. A lower concentration of toxic metabolites translates into better tolerance of ethyl alcohol, its higher consumption, and a potentially higher risk of developing addiction [30,31]. As mentioned above, the rs1693482 polymorphism remains in linkage disequilibrium with the rs1789891 polymorphism, i.e., the C allele of the *ADH1B/1C* rs1789891 gene is correlated with the C allele of rs1693482. The same is true for the T allele of the polymorphism that we studied (rs1789891). Therefore, people who carry the C allele of the rs1789891 polymorphism metabolize alcohol faster, in contrast to people who carry the A allele (rs1789891) [31,36]. The role of rs1789891 is inconclusive. There are studies that do not support the association between rs1789891 and the risk of alcohol dependence [37], while other authors point to an association between carrying the A allele of this polymorphism and a greater risk of alcohol dependence [30]. 

The third SNP of interest is rs3813867 of the *CYP2E1* gene. The CYP2E1 enzyme encoded by this gene belongs to the group of CYP450 enzymes that are responsible for the metabolism of numerous xenobiotics, including drugs and ethyl alcohol [20,38]. In this polymorphism, guanine (C1 allele) is converted to cytosine (C2 allele) at position 1259 of the long arm of chromosome 10. The C2 allele corresponds to cytosine, while the C1 allele is bound to guanine [39,40]. The transcriptional activity of the *CYP2E1* gene is increased 10-fold in carriers of the C2 allele [41]. It is also important to note that chronic alcohol consumption is an inducer of CYP2E1 enzyme activity, which can lead to the synthesis of toxic metabolites [42]. CYP2E1 also generates free radicals when ethanol is metabolized [41,43]. Regarding ethanol, studies suggest that carrying the C2 allele is associated with increased alcohol consumption [44]. Researchers also suggest that *CYP2E1* polymorphisms may play a role in the pathogenesis of FASD [45,46]. The newborn’s ability to eliminate alcohol averages 83.5% of the mother’s rate. Many studies have reported cases in which the blood alcohol content (BAC) of the newborn after birth was higher than the BAC of the mother. This can be explained not only by the lower ability of the fetus to eliminate alcohol but also by the phenomenon of the constant intake of amniotic fluid, which accumulates alcohol excreted by the fetus in unchanged form (due to low metabolic efficiency) [17]. The estimated efficiency of alcohol metabolism by the fetal liver is about 5–10% (of that reported in adults) in the early stages of development, mainly due to alcohol dehydrogenase, which is detected around the 8th week of pregnancy [47,48]. The ability of the fetal liver to metabolize alcohol increases with the duration of pregnancy, reaching a maximum of 50% of the maternal liver capacity [49]. On the other hand, the activity of CYP2E1 enzyme can be detected around the 19th week of pregnancy [50]. However, its activity is much lower than in adults [51,52]. The limited activity of ADH and CYP3A4 increases the toxicity of alcohol and its harmful metabolites, which may be further influenced by both the way the mother drinks and the mother’s and fetus’s genetically determined ability to metabolize ethyl alcohol [10]. The aim of this study is to answer the question of whether the SNPs of the *ADH1B*, *ADH1B/1C*, or *CYP2E1* genes in drinking mothers and children exposed to h-PAE have an impact on the risk of FASD and the degree of severity of FASD morphological features in the population of Polish children.

## 2. Materials and Methods

Throughout this paper, children with FAS are referred to as the FC group (FAS children) and other groups are referred to as children with pFAS—PFC (partial FAS children), or children without FAS—NFC (non-FAS children). Children in the number of 303 (213 girls, mean age 8.59 ± 4.77 years; 90 boys, mean age 9.77 ± 5.14 years) and 251 mothers (mean age 32.80 ± 5.92 years) were included in this study. The study group consisted of 303 children with hPAE, of whom 183 were diagnosed with FASD (partial FAS children—PFC, *n* = 42:37 girls, mean age 8.32 ± 3.92 years; 5 boys, mean age 10.60 ± 3.13 years, and FAS children—FC, *n* = 141:84 girls, mean age 8.43 ± 5.39 years; 57 boys, mean age 9.75 ± 4.48 years) and NFC children without the presence of obvious morphological FAS changes, unless there was a positive history of hPAE (non-morphological FAS children—NFC, *n* = 120:92 girls, mean age 8.58 ± 4.51 years; 28 boys, mean age 9.64 ± 6.62 years). The mother’s study group consisted of 251 women, mean age 32.77 ± 5.93, with confirmed alcohol consumption during pregnancy. Mothers were divided into subgroups accordingly to their children’s hPAE status. The demographics of the study group are summarized in Table 1.

All the children enrolled in this study met the criteria for the occurrence of high levels of alcohol exposure during fetal life. PAE was confirmed by the mother’s medical history or by an objectively documented background investigation (e.g., mother’s stay in a sobering center or detoxification unit during pregnancy). Detailed information regarding FASD and ADHD diagnosis enclosed in supplementary materials. The criteria for excluding children from this study were psychiatric disorders including significant mood and anxiety disorders requiring pharmacological treatment, psychotic disorders (current and history), risky and harmful use of psychoactive substances (SPAs) (current and history) or addiction to SPAs, and developmental disorders including autism spectrum disorder, profound and significant mental retardation, genetic diseases, severe and uncompensated somatic diseases (endocrinological, cardiovascular, renal, neoplastic, autoimmune, anorexia), and organic diseases with clinical manifestations (epilepsy), slight underweight and low height for age but within normal limits (>10th centile). A history of depressive episodes and emotional and behavioral disorders did not exclude patients from this study. 

### 2.1. Genetic Analysis

Genomic DNA was extracted from buccal swabs. Cells from the mucosal membrane were collected using a sterile synthetic swab. Prior to isolation, swabs were stored in a preservative buffer at +4 °C. DNA extraction was performed using the Genomic Micro AX SWAB Gravity Kit (A&A Biotechnology, Gdynia, Poland) according to the manufacturer’s protocol. Subsequently, based on spectrophotometric absorbance measurement (260/280 nm), the DNA was standardized to equal concentrations of 10 ng/µL. Genotyping for the following single nucleotide polymorphisms (SNPs), *ADH1B* rs1229984, rs1789891, *CYP2E1* rs3813867, was performed using prevalidated allelic discrimination TaqMan real-time PCR assays (TaqMan Assay ID’s: C___2688467_20, C___8829540_10, C___2431875_10, Life Technologies, Waltham, MA, USA) and TaqMan GTXpress Master Mix (Life Technologies, Waltham, MA, USA). All reactions were performed in duplicate in a final volume of 12 µL (reaction temperature profile: 95 °C, 20 s; 40 × 95 °C 1 s/60 °C, 20 s). Fluorescence data were measured using the ViiA7 Real-Time PCR System (Applied Biosystems, Waltham, MA, USA) after a 40-cycle reaction. Genotypes were assigned to individual samples after analysis using the TaqMan Genotyper software (Thermo Fisher Scientific, Waltham, MA, USA). Alleles correlated with extensive alcohol metabolism and presumably connected with high acetaldehyde production were considered as risk alleles in multivariate analysis.

### 2.2. Statistical Analysis

The χ2-Pearson test (chi2) and/or Fisher’s exact test were used to compare qualitative variables between the genotype groups, alleles, and data within genotype groups. Hardy–Weinberg equilibrium was calculated for genotype and allele distribution frequencies in each study group. Student’s *t*-test was used to calculate SNPs additive level. *p* value < 0.05 was considered statistically significant. Statistical analysis was performed using STATISTICA PL, ver. 13.1, software (StatSoft, Inc. 2016, Tulsa, OK, USA).

## 3. Results

The group of 303 children was divided into three subgroups: FAS children (FC), pFAS children (PFC), and non-FASD children (NFC). Mothers were divided into three groups: mothers of FAS children (FC mothers), mothers of pFAS children (PFC mothers), and mothers of non-FASD children (NFC mothers). The discrepancy in the number of mothers is due to the fact that some of the mothers who participated in this study were mothers of multiple children. Mothers were also counted for each group (FC, PFC, NFC). For example, a mother with three children with pFAS alone is counted as one. Conversely, mothers with both FAS and non-FASD children were counted as FC and NFC mothers. First, the frequency of genotypes in mothers and children was compared separately for FAS and pFAS and no statistically significant differences were found against non-FAS (Supplementary Tables S1–S4). Then, in order to increase the statistical power of the conducted analysis, the FAS and pFAS groups were combined and again compared with non-FAS mothers (Table 2) and children (Table 3). The analysis conducted showed no differences in the frequency of genotypes. For all the analyses shown in Table 2 and Table 3, and the Supplementary Tables, the genotype distribution was in concordance with Hardy–Weinberg Equilibrium (HWE, *p* > 0.05). Moreover, the results of our study did not confirm that the infants are more likely to develop FASD if their and their mothers’ genotypes have a specific combination for an additive effect (Supplementary Table S5).

## 4. Discussion

This study examined the association between the polymorphisms of the genes encoding *ADH* and *CYP3A4* and the risk of FASD in children exposed to ethanol in the prenatal period. There was no association between the polymorphisms of the genes *ADH1b* (rs1229984), *ADH1B/1C* (rs1789891), and *CYP2E1* (rs3813867) and the risk of FASD.

### 4.1. ADH1B

The first of the studied polymorphisms of the *ADH1B* gene (rs1229984) has been evaluated in the context of the pathogenesis of alcohol use disorder (AUD). Genetic variation within the *ADH1B* gene determines the rate at which ethanol is metabolized to acetaldehyde [53,54]. The conversion of Arg48 to His48 is the effect of substituting adenine (A) for guanine (G). Two alleles are therefore distinguished: the allele (G) encoding arginine at position 48, which is considered the most common allele, and the allele (A) encoding histidine at position 48, which is more common in East Asian populations [19,26,53,54]. This change (Arg48His) leads to an approximately 40-fold increase in the enzymatic activity of alcohol dehydrogenase, and, as a result, the negative effects of alcohol consumption—for example, nausea or abdominal pain—are felt more quickly [19]. Thomasson et al. showed significant differences in the frequency of genotypes and the A allele of the rs1229984 between addicts and non-alcoholics. In the former, the A allele occurred much less frequently. In accordance with this, individuals who were homozygous for the AA genotype were less likely to meet the DSM-3 criteria for alcohol dependence compared to individuals who were heterozygous for the GA genotype or homozygous for the GG genotype [55]. The protective role of the A rs1229984 allele in relation to alcohol dependence was further supported by the work of Thomasson et al. and many other studies [26,27,56,57,58,59].

Less is known about the impact of the *ADH1B* gene SNP (rs1229984) in the development of FASD. In 2001, a study by Viljoen et al. showed a lower incidence of the A allele in mothers and their children with FAS. The study was conducted on 56 mother–child pairs with FAS. A possible explanation for this phenomenon is the protective role of this allele, which is associated with faster metabolism of ethanol to acetic aldehyde, resulting in the aforesaid increased symptoms of alcohol intolerance and thus less propensity to consume alcohol [60].

The protective effect of the A allele of the *ADH1B* (rs1229984) gene in the context of FASD appears plausible. Its carriers metabolize alcohol faster, which determines the lower exposure of the fetus to its harmful effects. In addition, a lower risk of addiction in the carriers may prevent alcohol dependence and thus indirectly protect the child from fetal exposure to alcohol. In addition, Zuccolo et al. showed that mothers with the A allele drank less alcohol during pregnancy, were less likely to binge-drink, and were more likely to quit drinking during pregnancy. Indirectly, this indicates the potential protective role of the allele by reducing the propensity of the mother to drink alcohol during pregnancy [30,61].

However, the results of the current study showed no protective effect of the *ADH1B* polymorphism (rs1229984) on the risk of FAS or pFAS (Table 2 and Table 3). In the case of children, the analysis of genotypes did not show any association between PFC + FC vs. NFC (*p* = 0.436). We obtained a comparable outcome for the mothers’ genotypes (*p* = 0.438). No significant results were also seen for individual analysis of FC vs. NFC, PFC vs. NFC—in both mothers and children’s groups (Supplementary Tables S1–S4). One of the limitations of the current study is a very low frequency of *ADH1B* variant alleles and genotypes in both mother’s and children’s groups (A allele frequency <5%, which resulted in only 1 AA homozygote among mothers, and the absence of AA homozygotes in children). That fact makes it difficult to reliably assess the effect of the rs1229984 SNP. Similarly, in other studies on European populations or of populations of European descent (e.g., Australian), the low frequency of this polymorphism is pointed out as a significant obstacle in assessing its role in FASD pathology [30,53,57].

### 4.2. ADH1B/1C

The second genetic polymorphism tested was SNP rs1789891 located in the *ADH1B/1C* gene cluster. As already mentioned, this is a polymorphism that is in complete linkage with the SNP of the *ADH1C* rs1693482 gene [29,30]. The rs1789891 polymorphism studied in this paper is characterized by the presence of the C allele, which is considered to be potentially protective against alcohol dependence, and the A allele, which exerts no such effect [31,36]. The choice of that polymorphism resulted from its still unexplained role in the development of FASD, despite attempts to assess its importance in predisposing to alcohol dependence. Bach et al. showed that carrying the A allele is associated with a higher risk of relapse to ethyl alcohol dependence, and that A allele carriers consume more of it than people with the homozygous CC genotype [36]. In addition, the same study demonstrated reduced gray matter loss in the CC homozygotes [36]. This may indicate an indirect neuroprotective effect of this allele, and thus, in the context of alcohol exposure in fetal life, may protect the nervous system of the developing fetus. The GWAS study by Frank et al. also suggests a protective role of the C allele in the context of alcohol dependence [27]. Similar results were found by Way et al. [28].

Despite the potential importance of the polymorphism, such studies have not yet been conducted on a group of children exposed to high levels of alcohol during their fetal period. The results of the current research revealed no association between rs1789891 and FASD risk. Mothers’ genotype did not impact the risk of FAS/pFAS (*p* = 0.587) and neither did the children’s genotype (*p* = 0.954) (Table 2 and Table 3). Individual genotype comparisons for FC vs. NFC and PFC vs. NFC did not show statistically important results (Supplementary Tables S1–S4). The frequency of the rs1789891 alleles and genotypes was similar in all study groups, with the CC genotype being the most common. As the SNP does not directly affect enzymatic activity, and the linkage varies among populations, it is hard to predict the effect of the SNP on alcohol metabolism in the study subjects. Hence, it is difficult to identify the cause of the negative results of the SNP association with the risk of FASD. In the population under study, the rs1789891 SNP did not appear to play a significant role in the risk of fetal alcohol syndrome in children exposed to hPA, even though the C allele was previously pointed out as potentially protective against alcohol dependence. In future research, other *ADH1* variants may be investigated, including the aforementioned rs1693482 polymorphism of the *ADH1C* gene. 

### 4.3. CYP2E1

CYP2E1 is a major component of the P-450 cytochrome, which plays an important role in the metabolism of ethyl alcohol [62]. The activity of CYP2E1 is genetically determined, which is important because CYP2E1 activity tends to increase in people who abuse alcohol [63]. Therefore, the last polymorphism we studied was the SNP of the cytochrome P-450 2E1 (*CYP2E1*) gene—rs3813867. This polymorphism was associated with oral and gastric cancer [64,65]. It might be a result of the increased production of free oxygen radicals during the metabolism of some xenobiotics in reactions catalyzed by cytochrome CYP2E1 [63]. 

Cytochrome P450 2E1 has also been examined in numerous studies on liver diseases [66,67,68], the metabolism of xenobiotics [69,70,71], or the side effects of various drugs, including those used to treat tuberculosis [72,73]. Within the described polymorphism, the presence of two alleles has been demonstrated, i.e., the c1 wild-type allele (G allele), which determines the lower enzymatic activity of CYP2E1 [41,74]. The second allele is the C2 allele (C allele), the presence of which is associated with a greater amount of CYP2E1 enzyme and thus a greater ability to metabolize xenobiotics, including ethanol [63]. Studies suggest that carrying the C2 allele is associated with increased alcohol consumption [44,75]. This is supported by research conducted by Konishi et al., who showed that men of Mexican-American origin who carried the C2 allele had a higher risk of alcohol dependence [76]. It is worth mentioning, in the context of the role of this polymorphism—which contributes to more toxic metabolites of alcohol—that the presence of this polymorphism (C2 allele) is associated with a greater risk of alcoholic cirrhosis [77]. It is possible that *CYP2E1* polymorphisms may be involved in the pathogenesis of FASD [47]. This claim seems to be supported by the fact that in addition to the occurrence of this enzyme in the maternal liver, it is also found in the placenta, fetal liver, and fetal brain [78]. Therefore, it can be potentially expected that factors increasing CYP2E1 activity, such as the polymorphism rs3813867, may enhance oxidative stress through increased metabolism of ethyl alcohol, thus contributing to the development of FASD [18,79,80,81]. In addition, Lee et al. showed in an experimental model using human hepatocytes that placental human chorionic somatomammotropin (hCS) leads to CYP2E1 enzyme induction [82]. Concluding from that study, as well as from the fact that alcohol is also an inducer of CYP2E1, in the mother who consumes alcohol during pregnancy, the induction of CYP2E1 is more intense [68]. As polymorphism rs3813867 investigated in our study was previously associated with increased CYP2E1 hepatic content, one could expect that the combination of the presence of the C2 allele and alcohol-induced induction of CYP2E1 activity could be associated with an increased risk of FASD by intensifying the pathogenic process. This would be similar to adults, where the increased level of this enzyme associated with the presence of the C2 allele contributes to a higher risk of liver cirrhosis.

The results of the current research showed no association between the presence of the C2 allele and the risk of FAS (*p* = 0.262) or p-FAS (*p* = 0.678) in children exposed to high levels of fetal alcohol exposure (Supplementary Tables S1 and S2). Mothers’ genotypes also did not influence the risk of FAS and pFAS, *p* = 0.839 and *p* = 0.608, respectively.

Statistical analyses did not show any significant results for combined groups of PFC and FC children and mothers of PFC and FC, *p* = 0.475 and *p* = 0.724, respectively (Table 2 and Table 3). However, although the association was not confirmed, the potential importance of the studied polymorphism in the pathogenesis of FASD cannot be excluded, as the results might have been affected by the low allele frequency of this allele in the population. The C2 allele (corresponding to cytosine) constituted only 2.13% of the alleles in FC mothers, 1.22% in PFC mothers, and 2.10% in NFC mothers, whereas in children it was 2.13% (FAS), 4.76% (p-FAS), and 3.75% (non-FASD). In our study, none of the mothers or children, regardless of whether they had FAS, p-FAS, or non-FASD, were found to be homozygous for the c2 allele. Studies on another or bigger population may potentially yield different results consistent with theoretical considerations of the role of this polymorphism in the pathogenesis of FASD [45].

In summary, future studies may aim to study a more genetically diverse population, investigate other polymorphisms affecting alcohol metabolism, consider other risk factors, e.g., environmental, and relate these to the relevant genotypes.

The limitation of the present study is the low genetic diversity of the study population and the lack of consideration of other factors influencing the development of FASD, e.g., maternal comorbidities, body weight, maternal age, and the drinking pattern during pregnancy.

## 5. Conclusions

The results presented in this study do not support the hypothesis that SNPs within the *ADH1B* (rs1229984), *ADH1B* (rs1789891), and *CYP2E1* (rs3813867) genes have an effect on the risk of FAS and its degree (FAS or pFAS). The risk of FAS was not influenced by either maternal genotype or the child’s genotype. The strength of this study was a large group of subjects with confirmed hPE. The weakness, on the other hand, was the low genetic diversity of the study population. To the authors’ knowledge, this is the first study of polymorphisms of the *ADH1B/1C* (rs1789891) and *CYP2E1* (rs3813867) genes in the context of FASD that has attempted to experimentally associate the risk of FASD-related disorders with these polymorphisms.

## Figures and Tables

**Table 1:** Demographic data of patients (mothers and children) enrolled in the study.

hPAE Children Group	*n*	Age [Years]	Female Sex [*n*]	Female Sex [%]
total	303	8.94 ± 4.90	213	70.30%
FC	141	8.96 ± 5.07	84	59.57%
PFC	42	8.60 ± 3.87	37	88.07%
NFC	120	9.03 ± 5.06	92	76.67%
**Mothers Group**	***n***	**Age [Years]**		
total	251	32.77 ± 5.93		
total (hPAE groups) *				
FC	135	33.36 ± 5.97		
PFC	41	33.15 ± 4.53		
NFC	119	32.63 ± 5.91		

hPAE—high prenatal alcohol exposure, FAS children—FC, partial FAS children—PFC, non-morphological FAS children—NFC; * including mothers with more than one additional child.

**Table 2:** Genotypes and alleles in FC + PFC and NFC mothers in *ADH1B* rs1229984, rs1789891, and *CYP2E1* rs3813867.

	FC + PFC Mothers (*n* = 176)		NFC Mothers (*n* = 119)		*p* ^a^		*p* ^b^	OR (95% CI)
	*n*	%	*n*	%				
***ADH1B* rs1229984**								
**genotype**								
GG	157	89.21%	107	89.92%		TT + CT vs. CC	1.00	1.08 (0.50–2.32)
GA	19	10.79%	11	9.24%	0.438	TT vs. CT + CC	0.40	-
AA	0	0.00%	1	0.84%		TT vs. CC	0.41	-
						CT vs. CC	0.85	1.18 (0.54–2.57)
						TT vs. CT	0.39	-
***ADH1B* rs1229984**								
**allele**								
G	333	94.60%	225	94.54%				
A	19	5.40%	13	5.46%		T vs. C	1.00	0.99 (0.48–2.04)
***ADH1B* rs1789891**								
**genotype**								
CC	108	61.36%	80	67.23%		AA + CA vs. CC	0.33	1.29 (0.79–2.11)
CA	58	32.96%	33	27.73%	0.587	AA vs. CA + CC	1.00	1.14 (0.40–3.21)
AA	10	5.68%	6	5.04%		AA vs. CC	0.80	1.24 (0.43–3.54)
						CA vs. CC	0.36	1.30 (0.78–2.18)
						AA vs. CA	1.00	0.95 (0.32–2.85)
***ADH1B* rs1789891**								
**allele**								
C	274	77.84%	193	81.09%				
A	78	22.16%	45	18.91%		A vs. C	0.35	1.22 (0.81–1.84)
***CYP2E1* rs3813867**								
**genotype**								
GG	170	96.59%	114	95.80%		CC + GC vs. GG	0.76	0.81 (0.24–2.70)
GC	6	3.41%	5	4.20%	0.724	CC vs. GC + GG	1.00	-
CC	0	0.00%	0	0.00%		CC vs. GG	1.00	-
						GC vs. GG	0.76	0.81 (0.24–2.70)
						CC vs. GC	1.00	-
***CYP2E1* rs3813867**								
**allele**								
G	346	98.30%	233	97.90%				
C	6	1.70%	5	2.10%		C vs. G	0.76	0.81 (0.25–2.68)

^a^ χ^2^ test. ^b^ Fisher’s exact test.

**Table 3:** Genotypes and alleles in FC + PFC and NFC children in *ADH1B* rs1229984, rs1789891, and *CYP2E1* rs3813867.

	FC + PFC (*n* = 183)		NFC (*n* = 120)		*p* ^a^		*p* ^b^	OR (95% CI)
	*n*	%	*n*	%				
***ADH1B* rs1229984**								
**genotype**								
GG	172	93.99%	110	91.67%		TT + CT vs. CC	0.49	0.70 (0.29–1.71)
GA	11	6.01%	10	8.33%	0.436	TT vs. CT + CC	1.00	-
AA	0	0.00%	0	0.00%		TT vs. CC	1.00	-
						CT vs. CC	0.49	0.70 (0.29–1.71)
						TT vs. CT	1.00	-
***ADH1B* rs1229984**								
**allele**								
G	355	97.00%	230	95.83%				
A	11	3.00%	10	4.17%		T vs. C	0.50	0.71 (0.30–1.71)
***ADH1B* rs1789891**								
**genotype**								
CC	122	66.67%	78	65.00%		AA + CA vs. CC	0.81	0.93 (0.57–1.51)
CA	54	29.51%	37	30.83%	0.954	AA vs. CA + CC	1.00	0.92 (0.28–2.95)
AA	7	3.82%	5	4.17%		AA vs. CC	1.00	0.90 (0.27–2.92)
						CA vs. CC	0.80	0.93 (0.56–1.55)
						AA vs. CA	1.00	0.96 (0.28–3.25)
***ADH1B* rs1789891**								
**allele**								
C	298	81.42%	193	80.42%				
A	68	18.58%	47	19.58%		A vs. C	0.75	0.94 (0.62–1.42)
***CYP2E1* rs3813867**								
**genotype**								
GG	173	94.54%	111	92.50%		CC + GC vs. GG	0.48	0.71 (0.28–1.81)
GC	10	5.46%	9	7.50%	0.475	CC vs. GC + GG	1.00	-
CC	0	0.00%	0	0.00%		CC vs. GG	1.00	-
						GC vs. GG	0.48	0.71 (0.28–1.81)
						CC vs. GC	1.00	-
***CYP2E1* rs3813867**								
**allele**								
G	356	97.27%	231	96.25%				
C	10	2.73%	9	3.75%		C vs. G	0.49	0.72 (0.29–1.80)

^a^ χ^2^ test. ^b^ Fisher’s exact test.

## Data Availability

Not applicable.

## Supplementary Materials

The following supporting information can be downloaded at: https://www.mdpi.com/article/10.3390/genes14071392/s1, Questionnaire developed by Wolańczyk and Kołakowski on the basis of DSM-IV Rating Scale (RS) and ICD-10 criteria; Supplementary Table S1. Genotypes and allele in FC and NFC children in ADH1B rs1229984, rs1789891, and CYP2E1 rs3813867; Supplementary Table S2 Genotypes and allele in PFC and NFC children in ADH1B rs1229984, rs1789891, and CYP2E1 rs3813867; Supplementary Table S3. Genotypes and allele in FC and NFC mothers in ADH1B rs1229984, rs1789891, and CYP2E1 rs3813867; Supplementary Table S4. Genotypes and allele in PFC and NFC mothers in ADH1B rs1229984, rs1789891, and CYP2E1 rs3813867.
