# Supplementary material for: ADH1B, ADH1B/C and CYP2E1 Gene Polymorphism and the Risk of Fetal Alcohol Spectrum Disorder
Source: Genes (Basel). 2023 Jul 2;14(7):1392. doi: 10.3390/genes14071392 (PMC10379323; doi:10.3390/genes14071392)
Supplement: Supplementary file 1 [file genes-14-01392-s001.zip › Supplementary Materials.pdf]

Supplementary materials

Genetically determined ethyl alcohol metabolism of *ADH1B*, *ADH1B/C* and *CYP2E1* and the risk of FASD

Arnold Kukowka[1,*] , Bogusław Brzuchalski[1] , Mateusz Kurzawski[2] , Damian Malinowski[1] and Monika Anna Białecka[1]

The clinical diagnosis of FASD was based on a 4-digit diagnostic questionnaire, according to a validation of the Polish version of the Washington Questionnaire for the Assessment of Fetal Alcohol Spectrum Disorders. All FASD children enrolled in the present study had severe CNS lesions or disturbed functionality not related to other genetic or environmental factors (both FC and PFC scored 3 or 4 on the 4th degree scale), significantly below average height or specific facial anomalies. According to the Washington Questionnaire, they were classified as generally moderate (3rd and 2nd degree) ARND. Of the total 303 children with hPAE enrolled in the study, 114 children met the criteria for a diagnosis of ADHD according to the American Psychiatric Association (APA) DSM-IV criteria and the ICD-10 Classification of Mental and Behavioral Disorders: Clinical Description and Diagnostic Guidelines. The quantitative assessment of the severity of symptoms was performed according to the questionnaire developed by Wolańczyk and Kołakowski based on the DSM-IV rating scale (RS) and ICD-10 criteria, which is not an international diagnostic valid instrument for ADHD screening but a very accessible and popular form of interview – simple in its interpretation in common practice (the template is available in the Supplementary Materials). It examines three areas of the child's dysfunction using separate scales: (I.) attention deficit which includes nine descriptions of problem behaviors, (II.) hyperactivity — five, (III.) impulsivity — four. The severity of each symptom is measured from 0 to 3 points (0 – the symptom never or very rarely occurs, 1 – sometimes, 2 – often, 3 – very often). Therefore, on each of the scales of the examined area, the patient may score a maximum of 27 points on the I. scale, 15 points on the II. Scale, and 12 points on the III. Scale. According to the DSM-IV RS criteria, the children under study were divided into 3 subgroups (subtypes) according to the predominance of the clinical picture (attention deficit disorder vs. activity/impulsivity assessed together): attention deficit/hyperactivity disorder (ADHD-A), hyperactivity/hyperimpulsivity disorder (ADHD-HI), and a mixed form (ADHD-M) when the criteria of both subtypes are balanced. The ICD-10 classification was used to study the contribution of the child's environmental functioning conditions to the clinical manifestation of hyperkinetic syndrome symptoms (F90.0/ADHD), taking into account all probable factors involved in its pathogenesis, except for the biological pathways of catecholamines involved in it. According to the diagnostic criteria, the F90.0 child's impaired functioning involves at least 2 of the 3 domains examined, including peer group, school, and home, observed simultaneously by at least 2 of the 3 assessments from the perspectives of different people in the child's environment.

Supplementary Table S1. Genotypes and allele in FC and NFC children in *ADH1B* rs1229984, rs1789891 and *CYP2E1* rs3813867.

	FC (n=141)		NFC (n=120)					
	n	%	n	%	p^a		p^b	OR (95% CI)
***ADH1B* rs1229984 genotype**								
CC	133	94.33%	110	91.67%		TT+CT vs CC	0.47	0.66 (0.25-1.73)
CT	8	5.67%	10	8.33%	0.398	TT vs CT+CC	1.00	-
TT	0	0.00%	0	0.00%		TT vs CC	1.00	-
						CT vs CC	0.47	0.66 (0.25-1.73)
						TT vs CT	1.00	-
***ADH1B* rs1229984 allele**								
C	274	97.16%	230	95.83%				
T	8	2.84%	10	4.17%		T vs C	0.47	0.67 (0.26-1.73)
***ADH1B* rs1789891 genotype**								
CC	97	68.79%	78	65.00%		AA+CA vs CC	0.60	0.84 (0.50-1.41)
CA	39	27.66%	37	30.83%	0.807	AA vs CA+CC	1.00	0.85 (0.24-2.99)
AA	5	3.55%	5	4.17%		AA vs CC	0.76	0.80 (0.23-2.88)
						CA vs CC	0.58	0.85 (0.49-1.45)
						AA vs CA	1.00	0.95 (0.25-3.55)
***ADH1B* rs1789891 allele**								
C	233	82.62%	193	80.42%				
A	49	17.38%	47	19.58%		A vs C	0.57	0.86 (0.55-1.35)
***CYP2E1* rs3813867 genotype**								
GG	135	95.74%	111	92.50%		CC+GC vs GG	0.30	0.55 (0.19-1.59)
GC	6	4.26%	9	7.50%	0.262	CC vs GC+GG	1.00	-
CC	0	0.00%	0	0.00%		CC vs GG	1.00	-
						GC vs GG	0.30	0.55 (0.19-1.59)
						CC vs GC	1.00	-
***CYP2E1* rs3813867 allele**								
G	276	97.87%	231	96.25%				
C	6	2.13%	9	3.75%		C vs G	0.30	0.56 (0.20-1.59)

[a] χ^2 test

[b] Fisher's exact test

Supplementary Table S2. Genotypes and allele in PFC and NFC children in *ADH1B* rs1229984, rs1789891 and *CYP2E1* rs3813867.

	PFC (n=42)		NFC (n=120)					
	n	%	n	%	*p*[a]		*p*[b]	OR (95% CI)
***ADH1B* rs1229984 genotype**								
CC	39	92.86%	110	91.67%		TT+CT vs CC	1.00	0.85 (0.22-3.24)
CT	3	7.14%	10	8.33%	0.807	TT vs CT+CC	1.00	-
TT	0	0.00%	0	0.00%		TT vs CC	1.00	-
						CT vs CC	1.00	0.85 (0.22-3.24)
						TT vs CT	1.00	-
***ADH1B* rs1229984 allele**								
C	81	96.43%	230	95.83%				
T	3	3.57%	10	4.17%		T vs C	1.00	0.85 (0.23-3.17)
***ADH1B* rs1789891 genotype**								
CC	25	59.52%	78	65.00%		AA+CA vs CC	0.58	1.26 (0.61-2.60)
CA	15	35.72%	37	30.83%	0.817	AA vs CA+CC	1.00	1.15 (0.22-6.16)
AA	2	4.76%	5	4.17%		AA vs CC	1.00	1.25 (0.23-6.84)
						CA vs CC	0.56	1.27 (0.60-2.68)
						AA vs CA	1.00	0.99 (0.17-5.66)
***ADH1B* rs1789891 allele**								
C	65	77.38%	193	80.42%				
A	19	22.62%	47	19.58%		A vs C	0.53	1.20 (0.66-2.19)
***CYP2E1* rs3813867 genotype**								
GG	38	90.48%	111	92.50%		CC+GC vs GG	0.74	1.30 (0.38-4.46)
GC	4	9.52%	9	7.50%	0.678	CC vs GC+GG	1.00	-
CC	0	0.00%	0	0.00%		CC vs GG	1.00	-
						GC vs GG	0.74	1.30 (0.38-4.46)
						CC vs GC	1.00	-
***CYP2E1* rs3813867 allele**								
G	80	95.24%	231	96.25%				
C	4	4.76%	9	3.75%		C vs G	0.75	1.28 (0.39-4.28)

[a] χ^2 test

[b] Fisher's exact test

Supplementary Table S3. Genotypes and allele in FC and NFC mother's in *ADH1B* rs1229984, rs1789891 and *CYP2E1* rs3813867.

	FC Mother's (n=135)		NFC Mother's (n=119)					
	**n**	**%**	**n**	**%**	***p*[a]**		***p*[b]**	**OR (95% CI)**
***ADH1B* rs1229984 genotype**								
CC	123	91.11%	107	89.92%		TT+CT vs CC	0.83	0.87 (0.38-2.02)
CT	12	8.89%	11	9.24%	0.562	TT vs CT+CC	0.47	-
TT	0	0.00%	1	0.84%		TT vs CC	0.47	-
						CT vs CC	1. 00	0.95 (0.40-2.24)
						TT vs CT	1.00	-
***ADH1B* rs1229984 allele**								
C	258	95.56%	225	94.54%				
T	12	4.44%	13	5.46%		T vs C	0.68	0.81 (0.36-1.80)
***ADH1B* rs1789891 genotype**								
CC	81	60.00%	80	67.23%		AA+CA vs CC	0.24	1.37 (0.82-2.29)
CA	47	34.82%	33	27.73%	0.465	AA vs CA+CC	1.00	1.03 (0.34-3.16)
AA	7	5.18%	6	5.04%		AA vs CC	1.00	1.15 (0.37-3.58)
						CA vs CC	0.22	1.41 (0.82-2.42)
						AA vs CA	0.77	0.82 (0.25-2.66)
***ADH1B* rs1789891 allele**								
C	209	77.41%	193	81.09%				
A	61	22.59%	45	18.91%		A vs C	0.33	1.25 (0.82-1.93)
***CYP2E1* rs3813867 genotype**								
GG	130	96.30%	114	95.80%		CC+GC vs GG	1.00	0.88 (0.25-3.11)
GC	5	3.70%	5	4.20%	0.839	CC vs GC+GG	1.00	-
CC	0	0.00%	0	0.00%		CC vs GG	1.00	-
						GC vs GG	1.00	0.88 (0.25-3.11)
						CC vs GC	1.00	-
***CYP2E1* rs3813867 allele**								
G	265	98.15%	233	97.90%				
C	5	1.85%	5	2.10%		C vs G	1.00	0.88 (0.25-3.08)

[a] χ^2 test

[b] Fisher's exact test

Supplementary Table S4. Genotypes and allele in PFC and NFC mother's in *ADH1B* rs1229984, rs1789891 and *CYP2E1* rs3813867.

	PFC Mother's (n=41)		NFC Mother's (n=119)					
	n	%	n	%	p^a		p^b	OR (95% CI)
***ADH1B* rs1229984 genotype**								
CC	34	82.93%	107	89.92%		TT+CT vs CC	0.27	1.84 (0.67-5.03)
CT	7	17.07%	11	9.24%	0.337	TT vs CT+CC	1.00	-
TT	0	0.00%	1	0.84%		TT vs CC	1.00	-
						CT vs CC	0.25	2.00 (0.72-5.57)
						TT vs CT	1.00	-
***ADH1B* rs1229984 allele**								
C	75	91.46%	225	94.54%				
T	7	8.54%	13	5.46%		T vs C	0.30	1.62 (0.62-4.20)
***ADH1B* rs1789891 genotype**								
CC	27	65.85%	80	67.23%		AA+CA vs CC	1.00	1.06 (0.50-2.25)
CA	11	26.83%	33	27.73%	0.862	AA vs CA+CC	0.70	1.49 (0.35-6.24)
AA	3	7.32%	6	5.04%		AA vs CC	0.69	1.48 (0.35-6.33)
						CA vs CC	1.00	0.99 (0.44-2.22)
						AA vs CA	0.68	1.50 (0.32-7.03)
***ADH1B* rs1789891 allele**								
C	65	79.27%	193	81.09%				
A	17	20.73%	45	18.91%		A vs C	0.75	1.12 (0.60-2.10)
***CYP2E1* rs3813867 genotype**								
GG	40	97.56%	114	95.80%		CC+GC vs GG	1.00	0.57 (0.06-5.03)
GC	1	2.44%	5	4.20%	0.608	CC vs GC+GG	1.00	-
CC	0	0.00%	0	0.00%		CC vs GG	1.00	-
						GC vs GG	1.00	0.57 (0.06-5.03)
						CC vs GC	1.00	-
***CYP2E1* rs3813867 allele**								
G	81	98.78%	233	97.90%				
C	1	1.22%	5	2.10%		C vs G	1.00	0.58 (0.07-5.00)

[a] χ^2 test

[b] Fisher's exact test

Supplementary Table S5. Additive model of risk alleles of *ADH1B* rs1229984, r s1789891 and *CYP2E1* rs3813867 in FC+PFC and NFC children mother's and FC+PFC and NFC children.

group	subgroup	number of risk alleles							*p* *
		0	1	2	3	4	total	mean ±SD	
mother's	FC+PFC	10	51	96	19	0	176	1.71 ± 0.74	0.431
	NFC	5	32	68	13	1	119	1.77 ± 0.73	
children	FC+PFC	5	50	115	13	0	183	1.74 ± 0.62	0.763
	NFC	4	34	70	10	2	120	1.77 ± 0.72	

ADH1B rs1229984 risk allele: T; rs1229984 allele C; *CYP2E1* rs3813867 allele C

* t-test
